# Sleep in lonely heroin-dependent patients receiving methadone maintenance treatment: longer sleep latency, shorter sleep duration, lower sleep efficiency, and poorer sleep quality

**DOI:** 10.18632/oncotarget.20061

**Published:** 2017-08-08

**Authors:** Hong-Jie Li, Bao-Liang Zhong, Yan-Min Xu, Jun-Hong Zhu, Jin Lu

**Affiliations:** ^1^ Affiliated Wuhan Mental Health Center (The Ninth Clinical School), Tongji Medical College of Huazhong University of Science & Technology, Wuhan, Hubei, China; ^2^ Department of Psychiatry, The First Affiliated Hospital of Kunming Medical University, Kunming, Yunnan Province, China

**Keywords:** sleep, loneliness, heroin dependence, methadone

## Abstract

Given the socially isolated status of Chinese heroin-dependent patients (HDPs) and the significant association between loneliness and sleep problem in the general population, the impact of loneliness on sleep of HDPs is potentially substantial. The study aimed to test whether loneliness is associated with poor sleep in terms of quantity and quality in a consecutive sample of Chinese HDPs receiving methadone maintenance treatment (MMT). The study participants were 603 HDPs of three MMT clinics in Wuhan, China. Data on socio-demographic and clinical characteristics were collected by a standardized self-administered questionnaire. Sleep outcomes included sleep latency, sleep duration, sleep efficiency, and sleep quality. We measured depressive symptoms, loneliness, and sleep quality by using Zung’s Self-rating Depression Scale, the single-item self-report of loneliness, and the Pittsburgh Sleep Quality Index, respectively. Multiple linear regression was used to examine whether loneliness is independently associated with sleep measures. After controlling for the confounding effects of potential socio-demographic and clinical variables, loneliness was significantly associated with longer sleep latency, shorter sleep duration, lower sleep efficiency, and poorer sleep quality. Loneliness may exacerbate sleep disturbance in Chinese HDPs of MMT clinics. Psychosocial interventions aimed at reducing loneliness in MMT clinics would improve the sleep of HDPs.

## INTRODUCTION

Sleep disturbance is highly prevalent among heroin-dependent patients (HDPs) [1-3]. A meta-analysis based on 13 studies with 1857 patients reported a 78.5% prevalence of sleep problem in HDPs [4]. Evidence from empirical studies has shown that, in HDPs, sleep disorder is significantly associated with addiction relapse, multiple drug use, and poorer quality of life [4-7]. Therefore, sleep problem is of significant clinical interest in addiction treatment practice. To date, there have been many studies investigating factors that are significantly associated with HDPs’ sleep problem [2-5, 8-11], however, nearly all these studies focused on unmodifiable factors of sleep disturbance among HDPs, such as unemployment, long length of heroin use, and a history of injecting heroin. By contrast, very few research has studied factors that can be treated or changed.

Loneliness is defined as “the unpleasant experience that occurs when a person’s network of social relations is deficient in some important way, either quantitatively or qualitatively” [12, 13]. In China, due to the strong social stigma towards persons with heroin addiction [14], HDPs often experience social isolation and loneliness [15]. Accumulating evidence from population-based studies have shown that loneliness is a significant risk factor for a variety of physical and mental health problems, including depression, dementia, increased blood pressure and sleep disturbance [16-18]. Recent studies further suggest that diminished sleep is a potentially pathway by which loneliness adversely affect health [18-20]. Because loneliness and sleep problem are preventable or treatable [16], a better understanding on how loneliness affects sleep would facilitate the development of measures to reduce the many harmful effects of loneliness.

To the best our knowledge, no studies have investigated the association between loneliness and sleep problem of HDPs. Importantly, because psychosocial services are not routinely provided in Chinese addiction treatment practice [21], untreated loneliness would have a greater negative effect on the sleep of Chinese HDPs if it is true that loneliness is significantly associated with sleep disturbance. This study set out to examine the impact of loneliness on sleep patterns in a sample of HDPs receiving methadone maintenance treatment (MMT). Because previous studies showed that lonely people spent much more time in bed but much less time sleeping and loneliness impaired daytime functioning [20, 22, 23], the hypothesis of this study was that loneliness would be associated with longer sleep latency, shorter sleep duration, lower sleep efficiency, and poorer sleep quality.

## RESULTS

In total, 603 HDPs completed the survey. Of the 603 participants, 412 (68.3%) were males, and the average age was 38.1 years (standard deviation [SD]=7.0, range=21-59). Their mean sleep latency, duration, efficiency, and quality score (PSQI) were 35.7 min. (SD=34.2), 9.0 hours (SD=2.3), 87.5% (SD=15.3), and 6.6 (SD=3.8), respectively. Numbers (percentage) of patients who felt lonely “never”, “seldom”, “sometimes”, “often”, and “always” were 7 (1.2%), 259 (43.0%), 160 (26.5%), 141 (23.4%), and 36 (6.0%), respectively. A total of 337 patients sometimes or more frequently felt lonely; the prevalence of loneliness was 55.9%. Detailed socio-demographic and clinical characteristics of these respondents are displayed in Table 1.

**Table 1:** Latency, duration, efficiency and quality of sleep according to socio-demographic and clinical characteristics of HDPs receiving MMT

Characteristics	*n*	Latency (min.)^#^	Unstandardized Beta	Duration (hours)^$^	Unstandardized Beta	Efficiency (%)^$^	Unstandardized Beta	Quality^#^	Unstandardized Beta
Gender									
Male	412	38.6±38.3**	6.737	8.7±2.1***	-1.065***	87.0±15.2	-1.803	6.7±3.6	-0.283
Female	191	29.4±22.2	Reference	9.6±2.7	Reference	88.5±15.5	Reference	8.7±2.1	Reference
Age (years)									
20-39	327	36.6±28.5**	3.722	9.1±2.3	0.623**	87.1±14.8	-2.271	6.6±3.7	-0.216
40-59	276	34.5±40.0	Reference	8.8±2.4	Reference	87.9±16.0	Reference	6.7±4.0	Reference
Education years									
<9	140	34.9±22.7	-2.437	9.3±2.4	0.525*	88.1±14.8	1.135	6.6±3.8	0.226
≥9	463	35.0±36.1	Reference	9.0±2.3	Reference	88.1±14.4	Reference	6.6±3.9	Reference
Marital status									
Married	295	34.4±38.4*	-1.249**	9.1±2.5	0.393	88.7±14.0	-0.558	6.4±3.7	-0.084
Non-married&	308	35.6±28.1	Reference	9.0±2.2	Reference	87.5±15.0	Reference	6.8±4.0	Reference
Employment									
Yes	271	34.3±38.1**	-1.432*	9.4±2.5**	0.547*	90.8±12.2***	5.355***	5.9±3.5***	-1.042**
No	332	36.7±28.7	Reference	8.7±2.2	Reference	85.6±16.2	Reference	7.4±4.0	Reference
Self-rated economic status&									
Good	149	34.0±42.4	1.023	8.9±2.1	-0.412*	91.2±13.3*	1.022	5.8±3.4**	-1.386
Fair	314	36.8±32.4	1.976	9.1±2.4	-0.525	87.0±14.5	0.542	6.8±3.7	-2.579
Poor	140	33.5±24.7	Reference	9.2±2.4	Reference	86.9±15.4	Reference	7.1±4.6	Reference
Route of heroin administration									
Smoking	96	25.6±22.3***	-9.849*	9.9±2.4***	1.004***	91.7±13.7**	2.783*	5.6±3.7***	-0.494**
Injecting	507	36.8±35.0	Reference	8.9±2.3	Reference	87.4±14.6	Reference	6.8±3.8	Reference
Duration of heroin use (years)									
<10	230	32.7±27.0**	-2.497*	9.4±2.3***	0.347	89.8±12.7***	4.481**	6.0±3.5***	-1.051**
≥10	373	38.5±41.5	Reference	8.5±2.3	Reference	85.3±16.6	Reference	7.5±4.2	Reference
Duration of MMT (months)									
<24	240	30.0±19.5	-2.211	9.2±2.2	0.168	89.2±13.8	1.668	5.9±3.4***	-0.836**
≥24	363	38.1±39.9	Reference	9.0±2.4	Reference	87.2±14.9	Reference	7.1±4.0	Reference
Methadone dosage (mg/day)									
<70	273	34.2±28.3	-0.187	9.0±2.1	-0.009	88.6±13.4	0.465	6.2±3.5	0.351
≥70	330	35.6±37.2	Reference	9.1±2.5	Reference	87.7±15.3	Reference	6.9±4.1	Reference
Depression									
Yes	204	44.1±37.3***	14.612***	9.1±2.3	0.177	86.5±15.0*	-0.641*	8.2±4.0***	2.391***
No	399	29.0±30.0	Reference	9.0±2.4	Reference	89.4±13.5	Reference	5.3±3.1	Reference
Loneliness									
Yes	337	37.4±31.9**	10.195**	8.8±2.3**	-0.611**	86.3±14.7**	-3.402*	7.4±3.9***	0.907**
No	266	32.7±35.4	Reference	9.4±2.3	Reference	90.2±14.0	Reference	5.6±3.5	Reference

^#^Mann-Whitney U test.

^$^*t*-test.

^&^one-way analysis of variance.

^&^“Non-married” included never-married, remarried, cohabitating, separated/divorced, and widowed.

*P < 0.05, **P < 0.01, ***P < 0.001.

As shown in Table 1, compared to not lonely patients, lonely patients had significantly longer sleep latency (z=3.111, P=0.002), shorter sleep duration (t=2.807, P=0.005), lower sleep efficiency (t=2.548, P=0.011), and higher PSQI score (t=2.752, P=0.006). Table 1 also shows that these sleep measures significantly differed between some subgroups according to socio-demographic and clinical characteristics, indicating that socio-demographic and clinical variables may bias the association between loneliness and sleep measures. After controlling for potential socio-demographic and clinical confounders, results of the multiple linear regression analyses (Table 1) reveal that loneliness remained significantly associated with longer sleep latency (unstandardized coefficient [Beta]=10.195, P=0.006), shorter sleep duration (Beta=-0.611, P=0.009), lower sleep efficiency (Beta=-3.402, P=0.037), and poorer sleep quality (Beta=0.907, P=0.008), respectively.

## DISCUSSION

In this study, the estimated prevalence of loneliness was 55.9% among Chinese HDPs of MMT clinics. In comparison with studies using similar definitions of loneliness, this prevalence is much higher than that reported in Chinese rural-to-urban migrant workers (18.3%) and older adults (33%) [13, 17]. The high prevalence of loneliness in HDPs found in this study is concordant with the significantly higher level of loneliness experienced by young MDMA users and individuals with substance use disorder compared with healthy controls [24, 25].

Previous studies have found that the sleep of methadone-maintained HDPs is characterized by longer latency, shorter duration, lower efficiency, more awakenings, shorter slow wave sleep, and poorer sleep quality [10, 26]. Our study revealed that, in methadone-maintained HDPs, lonely individuals relative to not lonely individuals had longer sleep latency, shorter sleep duration, lower sleep efficiency, and poorer sleep quality, and the influence of loneliness on sleep was independent of socio-demographic and clinical covariates. These findings suggest that lonely HDPs have more difficulties in initiating and maintaining sleep, and the two together result in poor sleep quality.

Endogenous opioid peptides play an important role in the onset and maintenance of sleep and sleep/wake transition [26, 27]. Because opioid receptors can also be activated by exogenous opiates [26], heroin suppresses the endogenous opioid systems in the brain. Due the feedback inhibition of endogenous opioid system caused by long-term use of heroin, HDPs may exhibit difficulties in sleep initiation and maintenance such as longer latency and shorter duration.

Existing studies on the impact of loneliness on sleep of the general population found that loneliness is significantly associated with daytime dysfunction, sleep fragmentation, and poor sleep quality, but not sleep duration [18-20]. It seems that loneliness does affect various measures of sleep other than the amount of sleep. Our findings are not fully consistent with results from these literature. Differences in subjects (older adults vs. HDPs) and measures of sleep (i.e., subjective vs. objective) may explain such discrepancy. However, it is also possible that loneliness has a more profound negative effect on sleep of HDPs, and thus leads to impairments in both quantity and quality of sleep. There are two possible reasons for the loneliness-sleep link. First, there is evidence that feelings of loneliness are associated with an increase in circulating cortisol and a high level of cortisol triggers the stress response [19, 28, 29], which in turn keeps lonely individuals awake at night. Second, the association between loneliness and poor sleep may also be explained by the heightened vigilance in lonely individuals, because people need a safe social surround to sleep soundly but lonely individuals are driven by increased vigilance for threat [19].

The present study has several limitations. Due to the cross-sectional nature of the study, we can not determine whether loneliness precedes sleep problem and infer the causal relationship between loneliness and sleep problem. Second, comparing the nocturnal cortisol levels of lonely and not lonely HDPs might help explain the loneliness-sleep link, but we did not measure this potentially useful biomarker due to deficiencies in the study design. Third, some other related factors of sleep problem (negative life events, lack of social support, etc.) were not evaluated in the study so our analysis is not able to exclude the confounding effects of these factors, which might influence the loneliness-sleep association. Finally, measures of sleep used in this study are subjective in nature, which are different from those collected objectively such as polysomnography. More studies are warranted to explore the impact of loneliness on objective measures of sleep.

In summary, there is a significant association between loneliness and poor sleep in terms of quality and quantity, including longer sleep latency, shorter sleep duration, lower sleep efficiency, and poorer sleep quality, indicating that loneliness may exacerbates sleep disturbance of HDPs. Given the high prevalence of loneliness in HDPs and the unavailability of psychosocial services in Chinese MMT clinics, providing psychosocial services to reduce or prevent loneliness might be an effective way to reduce sleep problem of methadone-maintained HDPs.

## MATERIALS AND METHODS

### Subjects

This cross-sectional study was conducted in three MMT clinics in Wuhan, China, between December 2009 and March 2010. A consecutive sample of adult patients who met DSM-IV criteria for lifetime heroin dependence and were taking methadone for drug rehabilitation in these MMT clinics, was invited to participate in this study. Patients with psychotic symptoms, serious physical illnesses, alcohol dependence, or brain organic mental disorders, were excluded.

The study protocol was approved by the Ethics Committee of Wuhan Mental Health Center. All participants provided written informed consent before the fieldwork of the survey.

### Instruments and procedures

This study used a self-administered questionnaire to collect data. Sleep quantity and quality in the past month were main outcomes of this study. The former included total hours of actual sleep at night (sleep duration), minutes taken to fall asleep each night (sleep latency), and the percentage of total actual sleep time out of the total time in bed (sleep efficiency). Sleep quality was measured with the Pittsburgh Sleep Quality Index (PSQI) [30], which is a widely used self-rated scale that assesses sleep quality and disturbances over the previous month. It has 19 items and each item is rated on a four-point integer scale (0-3). The total PSQI score is calculated by totaling the 19 item scores, with higher scores denoting a poorer sleep quality. Studies have proved the good reliability and validity of Chinese PSQI in both clinical and non-clinical settings [31-33].

Socio-demographic variables collected in the questionnaire were gender, age, education years, marital status, employment status, and self-rated economic status (good, fair, poor). Clinical characteristics collected included usual route of heroin administration (smoking, injecting), duration of heroin use, duration of MMT, methadone dosage, and depression. Depression was assessed with Zung’s Self-rating Depression Scale (SDS) [34]. The Chinese SDS is a 20-item self-rating scale to evaluate the severity of depressive symptoms and each item is scored on a 4-point scale (1=a little of the time, 2=some of the time, 3=good part of the time, 4=most of the time) [35]. Total SDS score varies between 20 and 80 and a cut-off score of 40 or greater is used to indicate clinically significant depression [34].

Loneliness was assessed by using a single question: “How often you feels lonely?”. The responses for this question were: 1=always, 2=often, 3=sometimes, 4=seldom, 5=never. This single-item measure of loneliness was widely used in prior studies [13, 36-38]. In line with prior studies [16, 36, 37], patients were classified as having loneliness if they felt lonely at least sometimes.

### Statistical analysis

Participants were divided into different subgroups (i.e,. male vs. female) according to their socio-demographic and clinical characteristics. Sleep measures of these subgroups were described and compared by t-test, one-way analysis of variance, or Mann-Whitney U test, as appropriate. The effect of loneliness on sleep was assessed with multiple linear regression that entered a sleep measure as the outcome variable, loneliness as the predictor, and socio-demographic and clinical covariates at once to adjust for the potential confounding effects of these socio-demographic and clinical variables. The statistical significance level was set at p<0.05 (two-sided). SPSS software version 15.0 package was used for analyses.
